# DNA Barcoding the *Dioscorea* in China, a Vital Group in the Evolution of Monocotyledon: Use of *mat*K Gene for Species Discrimination

**DOI:** 10.1371/journal.pone.0032057

**Published:** 2012-02-20

**Authors:** Xiao-Qin Sun, Ying-Jie Zhu, Jian-Lin Guo, Bin Peng, Ming-Ming Bai, Yue-Yu Hang

**Affiliations:** 1 Jiangsu Province Key Laboratory for Plant Ex Situ Conservation, Institute of Botany, Jiangsu Province and Chinese Academy of Sciences, Nanjing, People's Republic of China; 2 Institute of Medicinal Plant Development, Chinese Academy of Medical Sciences, Peking Union Medical College, Beijing, People's Republic of China; Biodiversity Insitute of Ontario - University of Guelph, Canada

## Abstract

**Background:**

*Dioscorea* is an important plant genus in terms of food supply and pharmaceutical applications. However, its classification and identification are controversial. DNA barcoding is a recent aid to taxonomic identification and uses a short standardized DNA region to discriminate plant species. In this study, the applicability of three candidate DNA barcodes (*rbc*L, *mat*K, and *psb*A-*trn*H) to identify species within *Dioscorea* was tested.

**Methodology/Principal Findings:**

One-hundred and forty-eight individual plant samples of *Dioscorea*, encompassing 38 species, seven varieties and one subspecies, representing majority species distributed in China of this genus, were collected from its main distributing areas. Samples were assessed by PCR amplification, sequence quality, extent of specific genetic divergence, DNA barcoding gap, and the ability to discriminate between species. *mat*K successfully identified 23.26% of all species, compared with 9.30% for *rbc*L and 11.63% for *psb*A-*trn*H. Therefore, *mat*K is recommended as the best DNA barcoding candidate. We found that the combination of two or three loci achieved a higher success rate of species discrimination than one locus alone. However, experimental cost would be much higher if two or three loci, rather than a single locus, were assessed.

**Conclusions:**

We conclude that *mat*K is a strong, although not perfect, candidate as a DNA barcode for *Dioscorea* identification. This assessment takes into account both its ability for species discrimination and the cost of experiments.

## Introduction

*Dioscorea* is a genus of more than 600 plant species in the family Dioscoreaceae [1], which contains approximately 70 sections. These species are mainly found in Southeast Asia, Africa, Central America, South America, and in other tropical or subtropical regions where some *Dioscorea* species are an economically important supply of starch in the staple diet. The genus is also a favored source of medicinal plants used to extract precursors of cortisone and other steroid hormones [2]–[4]. The importance of *Dioscorea* in terms of food supply and pharmaceutical use, together with the controversy over classification [5]–[8], has given impetus to improve the identification of this genus.

In this study, we aimed to establish a high-quality system for taxonomic identification to meet the requirements of agriculture and the pharmaceutical industry. Since the early 20^th^ century, morphology, cytology, palynology, and other traditional means of identification of this genus have been explored successively [9]–[16]. With the development of molecular biology, however, some DNA sequences, such as those of *rbc*L, *mat*K and *trn*L-F, have been used to solve complicated taxonomic problems and to infer phylogenetic relationships among organisms, including members of the Dioscoreaceae [17]–[19].

DNA barcoding has recently emerged as an aid for global species identification and has been successfully used in several studies when morphological characteristics were absent [20]–[23]. In animals, the mitochondrial gene cytochrome *c* oxidase I (*COI*) has provided a favorable solution in species identification [24]–[29]; however this gene has limited usefulness in plants. Therefore, several candidate regions have been proposed for use in plants, including portions of some coding genes (*mat*K, *rbc*L, *rpo*B and *rpo*C1) [23], noncoding spacers (*psb*A-*trn*H, *atpF*-*atp*H and ITS) [30]–[32], or a combination of several regions [33].

Little research has been carried out to investigate the applicability and effectiveness of different DNA regions as barcodes to identify species within *Dioscorea*. In particular, characterisation of species found in China, one of the most likely centers of origin [34], [35], was rarely included in previous studies. This study focuses on *Dioscorea* species distributed in China, and three candidate DNA barcode regions (*mat*K, *rbc*L and *psb*A-*trn*H) in the plastid genome were evaluated for identification. We aimed to address several questions: for example, which of these three regions is the most useful as a barcode and how effective are these three regions and their combinations for this discrimination?

## Results

### Sequence analysis and amplification efficiency

The sequence information of three candidate DNA barcode markers, *mat*K, *rbc*L and *psb*A-*trn*H, is provided in Table 1. For individual regions, aligned sequence lengths ranged from 535 bp for *psb*A-*trn*H to 752 for *mat*K. *rbc*L was the most conserved gene (522/553 nucleotides), based on both sequence length and number of conserved sites. *mat*K had the greatest nucleotide variation (110/752), followed by *psb*A-*trn*H (74/535), based on sequence length and number of variable sites. *psb*A-*trn*H had the highest percentage of parsimony (parsim)-informative sites (70/535), followed by *mat*K (81/752). It could be inferred that *psb*A-*trn*H and *mat*K are the best regions for use as DNA barcodes for phylogenetic reconstruction, whereas *rbc*L was the least suitable marker for *Dioscorea*.

**Table 1:** Sequence information of three candidate genes.

Marker	Sequence length (bp)	Alignment length (bp)	Conserved sites (bp)	Variable sites (bp)	Parsim-informative sites (bp)
*mat*K	794–1054	752	642	110	81
*rbc*L	631–743	553	522	31	23
*psb*A-*trn*H	268–631	535	454	74	70

The efficiency of PCR amplification is one of the important indicators for evaluating the applicability of DNA barcodes. The amplification efficiency of *mat*K using universal primers was 70.23% (the lowest efficiency found), while the amplification rate of *rbc*L and *psb*A-*trn*H was 83.33% and 74.07%, respectively. 81.4% amplification efficiency was achieved with another *mat*K universal primer set 3F_Kim/1R_KIM. Samples that failed to amplify with universal primers were successfully amplified using specific primer sets designed by ourselves based on the *Dioscorea* sequences available in Genbank. Samples that amplified successfully using universal primers were randomly picked to be amplified by the self-designed primers to verify their scope of use; amplification success rates were all found to be 100%.

### Intra-specific variation and inter-specific divergence

The maximum intra-specific divergence and the minimum inter-specific divergence of the three candidate barcodes and their combinations, *mat*K+*rbc*L, *mat*K+*psb*A-*trn*H, *rbc*L+*psb*A-*trn*H, *mat*K+*rbc*L+*psb*A-*trn*H were estimated using six metrics [36]. The non-coding region (*psb*A-*trn*H) showed greater intra- and interspecific divergence than the coding regions (*mat*K and *rbc*L; Table 2). *Psb*A-*trn*H had the highest interspecific divergence, followed by that of *rbc*L+*psb*A-*trn*H and *mat*K+*psb*A-*trn*H, and the inter-specific divergence of *rbc*L was the lowest (Table 2). *mat*K had the maximum intra-specific variation while *rbc*L had the minimum. Furthermore, all seven barcodes showed higher genetic variability between than within species.

**Table 2:** Measures of inter- and intra-specific divergence and identification efficiency of the potential barcodes and combined barcodes.

		matK	rbcL	psbA-trnH	matK+rbcL	matK+psbA-trnH	rbcL+psbA-trnH	matK+rbcL+psbA-trnH
All intra-specific distances		0.0095±0.0167	0.0019±0.0045	0.0195±0.0469	0.0062±0.01	0.0082±0.0127	0.0072±0.0147	0.0082±0.0127
Mean theta		0.0118±0.0164	0.0026±0.0044	0.0155±0.0273	0.0078±0.0098	0.0089±0.009	0.0065±0.009	0.0089±0.009
Average coalescent depth		0.0178±0.0224	0.005±0.0074	0.0401±0.0699	0.0121±0.0139	0.0161±0.017	0.0156±0.0221	0.0161±0.017
All interspecific distances		0.0295±0.0249	0.0125±0.0076	0.0879±0.0857	0.0222±0.0162	0.0325±0.0246	0.0355±0.0293	0.0325±0.0246
The minimum interspecific distance		0.0035±0.009	0.0013±0.0029	0.0022±0.0061	0.0029±0.0062	0.0031±0.006	0.002±0.0035	0.0031±0.006
Efficiency of PCR amplification with universal primers/%		70.23 (81.40)^1^	83.33	74.07				
Relative identification efficiency/%^2^	Blast1	23.26 (27.70)	9.30 (10.81)	11.63 (20.27)	46.51 (60.81)	32.56 (53.38)	37.21 (38.51)	53.49 (74.32)
	Nearest distance	23.26 (27.70)	9.30 (10.81)	4.65 (14.86)	46.51 (60.81)	30.23 (50.00)	27.91 (34.46)	53.49 (71.62)

1 Efficiency of PCR amplification with universal primer 3F_Kim and 1R_KIM recommended by CBOL (http://barcoding.si.edu) in a sample pool composed of one randomly selected sample from all species.

2 Species identification efficiency with sample identification efficiency in bracket.

### Statistical comparison of divergence

It can be seen from the Wilcoxon signed rank tests that the inter-specific divergence of *mat*K was higher than that of *rbc*L, and *rbc*L exhibited a higher inter-specific divergence than did *psb*A-*trn*H (Table 3). *P*-values were less than zero showed that the differences were highly significant. These statistically analysed data suggest that *mat*K would serve as an ideal candidate for identifying *Dioscorea*.

**Table 3:** Wilcoxon signed rank test of the inter-specific divergences among the three loci.

W+	W−	Relative Ranks, n, P value	Result
*mat*K	*rbc*L	W+ = 5949, W− = 3268, *n* = 10403, *P*≤0.0	*mat*K>*rbc*L
*mat*K	*psb*A-*trn*H	W+ = 2863, W− = 6319, *n* = 10368, *P*≤0.0	*mat*K>*psb*A-*trn*H
rbcL	*psb*A-*trn*H	W+ = 2503, W− = 6299, *n* = 10213, *P*≤0.0	*rbc*L>*psb*A-*trn*H

### DNA barcoding gap assessment

**Figure 1:**
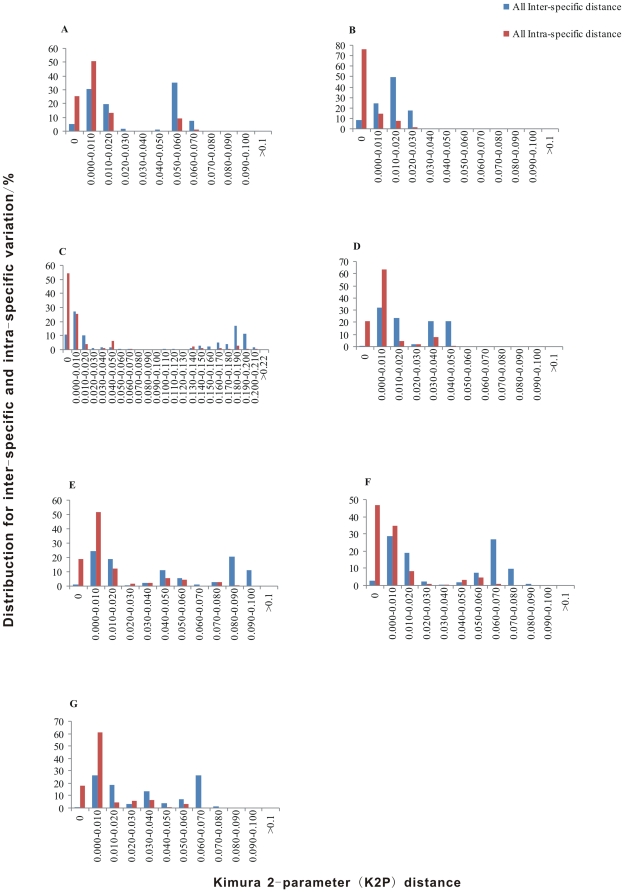
Relative distribution of inter-specific divergence between congenic species and intra-specific variation. (A) *mat*K. (B) *rbc*L. (C) *psb*A-*trn*H. (D) *mat*K+*rbc*L. (E) *mat*K+*psb*A-*trn*H. (F) *rbc*L+*psb*A-*trn*H. (G) *mat*K+*rbc*L+*psb*A-*trn*H.

We examined the distributions of intra-specific versus inter-specific divergence in the seven barcodes at a scale of 0.001 distance units. Although no distinct barcoding gaps as typical of *CO1* were found in the distributions of all the loci, it does suggest a clearly defined range, where the intraspecific variation is considerably lower than the interspecific divergence (Fig. 1). And among them, *mat*K revealed a relatively well separated distribution. For *mat*K, the intra-specific distances mainly distributed in section 0.000–0.010, while the inter-specific distances mainly distributed in section 0.050–0.060. And for *mat*K congeneric species with a genetic distance of zero accounted for only 4.914% of the total samples (8.209% for *rbc*L and 10.57% for *psb*A-*trn*H). So it's proposed that *mat*K could be used to discriminate most species in this study. The loci combination *mat*K+*rbc*L+*psb*A-*trn*H could also be used for species identification in *Dioscorea* with the lowest ratio of samples (0.486%) having an inter-specific distance of zero. For *mat*K+*rbc*L+*psb*A-*trn*H, the intra-specific distances mainly distributed in section 0.000–0.010, and the inter-specific distances mainly distributed in section 0.060–0.070. Furthermore, it was confirmed that the interspecific divergences of all the seven loci was significantly higher than that of the corresponding intraspecific variations by Wilcoxon two-sample tests. And the most significant difference was observed in *mat*K for single locus and *mat*K+*rbc*L+*psb*A-*trn*H for loci combination (Table S1).

### Applicability for species discrimination

BLAST1 searches and the nearest genetic distance were used to test the applicability of the three loci and four combinations for species identification (Table 2). Our results revealed that *mat*K possessed the highest identification efficiency of the three loci. In contrast, the rates of successful species identification using *psb*A-*trn*H were the lowest. In addition, the success rates of combined barcodes were higher than those of the single locus using both methods. *mat*K+*rbc*L+*psb*A-*trn*H had the highest authentication capability, which correctly identified 53.49% of the species by both the BLAST1 search and the nearest genetic distance methods.

## Discussion

### Assessment of the applicability of the three candidate barcodes

An ideal DNA barcode must have high PCR amplification efficiency, whilst containing enough variability to be used for species identification and adequate conserved regions for the design of universal primers [37]. In this study, it was found that the chloroplast *mat*K gene was a promising candidate for authenticating *Dioscorea* species based on amplification efficiency, barcoding gaps, and success rate of identification. An amplification efficiency of 100% was obtained using specific primers and the identification efficiency was highest when using the three loci (23.26%). The chloroplast *rbc*L gene did not have enough inter-specific divergence, although its amplification efficiency was not low. The success rate of identification of *psb*A-*trn*H was too low to be useful for this purpose.

*rbc*L and *psb*A-*trn*H had individual advantages despite their poor capability for authentication of *Dioscorea* species. *rbc*L had high amplification efficiency, but the overlap of intra-specific and inter-specific divergence was too substantial to be of use for discrimination and the identification rate was only 9.30%. This situation arises because the *rbc*L gene does not have sufficient variation at the species level to be use as a DNA barcode [31], [38]–[41]. Approximately 10,300 *rbc*L sequences in the GenBank were compared using the distance based method. It was found that *rbc*L was not capable of discriminating between all species, but was able to distinguish some taxa at the genus and species levels [42].

The amplification efficiency of *psb*A-*trn*H in *Dioscorea* was moderate and its identification accuracy was only 11.63%, therefore it is not a suitable candidate as a DNA barcode for *Dioscorea*, as it is for other species [43], [44]. In addition, the presence of a poly-A/T in this region often reduces the success rate of DNA sequencing.

Insertions or deletions appear to be a common characteristic of this genetic region, even in closely related species [45]–[50]. The variable lengths of this region make sequence alignment difficult. Large insertion or deletion was also found in different populations of *Dioscorea*. For example, *Dioscorea zingiberensis* C.H. Wright collected from Yichang, Madao and Enshi in China had a 234-bp insertion segment at 183 bp compared to other populations. The generation mechanism of indels in *psb*A-*trn*H remains ambiguous, and one hypothesis was raised by Aldrich et al., [45] that the deletion of insert often occurred between imperfect AT-rich repeats flanking the insert, which was also supported by the detection of imperfect AT-rich repeats flanking the indel in *Dioscorea zingiberensis*. In contrast with the problems of indels for sequence alignment, indels will ultimately enrich the information needed for species discrimination [30]. Such indel indicates a divergent trend of two groups separated by Yangtze River in *Dioscorea zingiberensis* [51]. The insertions were only detected in the three populations of north group, while the others exhibited deletion.

The combinations of *psb*K-*psb*I+*atp*F-*atp*H, *mat*K+*atp*F-*atp*H+*psb*K-*psb*I and *mat*K+*atp*F-*atp*H+*psb*A-*trn*H were used to discriminate 101 individual plants belonging to 31 species and 18 families, and achieved high success rate [40]. When combinations of two or three loci were used in *Dioscorea*, much higher identification efficiency was achieved than any of single locus.

### Application of *mat*K in discrimination of *Dioscorea* species

*mat*K is a recommended DNA barcode candidate gene because of its high evolution rate [23], [52], [53]. Lahaye and colleagues amplified *mat*K successfully in 398 samples using primers 390F/1326R in 2008 and more than 90% species could be identified. However, these results have been reviewed unfavorably by some researchers. Kress and colleagues [32] doubted whether amplification efficiency could remain high in plants from other families, as 96% of samples in Lahaye's study were orchids. The criticism of *mat*K for use as a DNA barcode is its poor performance of primer universality [52], [54] so different primers are needed to amplify samples from different taxa. Kress and Erickson [31] achieved a 39.3% amplification rate for 96 species belonging to 48 genera, although 10 pairs of primers were used. Fazekas and colleagues [39] amplified 251 individual plants from 32 genera and 92 species, but their success rate was only 87.6%.

The amplification efficiency of *Dioscorea* were 70.23% and 81.40% with primers intF/intR and 3F_Kim/1R_KIM respectively and a 100% success rate was achieved using our in-house designed specific primers. For an ideal barcode a distinct gap with no overlap is essential [23], [36]. But in this study, no distinct barcoding gap was found even though intra-specific divergence and interspecific divergence was mainly non-overlapping (Fig. 1). Nevertheless, based on the histogram of DNA barcoding gap and species identification, *mat*K was proven to be better than the other loci in our study.

ITS and ITS2 have been also proposed to be the most promising universal DNA barcode in plants [55], [56], unfortunately, because of the low sequencing success of the ITS and ITS2 region brought by serious endophyte interference in our study, this region was not included for further analysis.

In conclusion, our study shows that the *mat*K is a strong, although not perfect, candidate for *Dioscorea* identification. It remains necessary to carry out further research on other more variable DNA barcodes such as *psbK-psbI* and *atpF-atpH* in species identification of this genus.

## Materials and Methods

### Plant materials

Plant samples were collected from different locations in China and identified by one of our authors, Prof. Yueyu Hang. In total, 148 individual samples belonging to 38 species, seven variants and one subspecies, representing majority species (46/61) and all six sections distributed in China of this genus, were collected for further analysis. Fresh leaves were dried in silica gel at the time of collection. Voucher specimens were deposited in the herbarium at the Kunming Institute of Botany, Chinese Academy of Sciences (KIM) (Table S2).

### DNA extraction, amplification, sequencing

Genomic DNA was extracted following a cetyl trimethylammonium bromide (CTAB) protocol modified from Paterson et al. [57]. The universal primers intF and intR (RBG Edinburgh recommended), 1F and 724R [58], and psbAF and trnH2 [59] were used in the amplification of *mat*K, *rbc*L and *psb*A-*trn*H regions of the cpDNA respectively. As to these samples failed to amplify using universal primers, specific primers were designed with the aid of OLIGO primer design software (Molecular Biology Insights, Inc., Cascade, Colorado, USA), based on genus *Dioscorea* sequences deposited in the GenBank database. For example the *mat*K sequence of *D. alata* L. (AB040208), the *rbc*L sequence of *D. alata* L. (AY667098) and the *psb*A-*trn*H region of *D. elephantipes* (L'Her.) Engl. (EF380353.1) were used. In addition, the universal primer set 3F_Kim and 1R_KIM currently recommended by CBOL (http://barcoding.si.edu) was also adopted to evaluate the efficiency of PCR amplification in a sample pool composed of one randomly selected sample from all species. Detailed sequences of all the primers and reaction conditions are listed in Table S3.

Polymerase chain reaction (PCR) amplification of the three candidate barcodes was carried out using the following program: a premelt of 3 min at 94°C, followed by 35 cycles of 45 s denaturation at 94°C, 30 s annealing reaction at 53–58°C, and finally a 1.5 min 30 s extension at 72°C. Each 20-µl reaction mixture contained 30 ng of genomic DNA template, 2.5 mmol/L MgCl2, 1× Mg-free DNA polymerase buffer, 0.12 mmol/L dNTPs, 0.3 µmol/L each primer, 1 U *Taq* DNA polymerase. PCR products were examined electrophorectically using 0.8–1.2% agarose gels. Purification and bidirectional sequencing were completed by Beijing Genomics Institute (BGI) using the amplification primers.

### Sequence alignment and data analysis

Sequences were aligned and adjusted manually using Sequencer v.4.5 software (GeneCodes, Ann Arbor, MI, USA). The nucleotide sequence data of the three regions were deposited in the GenBank database (Table S2). All genetic distances were calculated using MEGA (4.0 Version) software.

Average intra-specific distance, mean theta and coalescent depth were calculated to determine intra-specific variation [36], [55], and average interspecific distance, theta prime and the minimum interspecific distance were calculated to determine interspecific divergence [36], [55], [60]. Wilcoxon signed-rank tests were performed as previously described [23], [31]. The distribution of intra-specific versus interspecific variability was evaluated by assessment of the presence of DNA barcoding gaps [31], [36]. Two methods of species identification, including BLAST1 protein similarity search and the nearest distance method, were carried out as described previously [61]. BLAST1 searches were conducted on a local reference library constructed for each region. The barcode sequence of each species was queried against the local library with the “blastn” command. The identity of a sample was based on the best hit and the E-value for the match must be lower than the cutoff value. In comparison, for the nerest genetic distance method, the identity of a sample was determined based on the subject sequence which has the smallest genetic distance and the distance must be less than a distance threshold. The traffic light approach was used to identify the combination of markers [62].The combination would have identification power as long as the sequences could be identified by any of the markers in combination, while the combination would be incapable of identifying sequences if none of the markers in combination could identified sequences successfully.

## Supporting Information

Table S1 Wilcoxon two-sample tests for distribution of intra- vs. inter-specific divergences.
(DOC)

Table S2 Samples for testing potential barcodes and accession numbers in GenBank.
(DOC)

Table S3 Primers and reaction conditions used in the study.
(DOC)

## References

[1] Coursey DG (1967). Yams: an account of the nature, origins, cultivation, and utilization of the useful members of *Dioscoraceae*.

[2] Fujii K, Matsukawa T (1936). Saponins and sterols. 8. Saponin of *Dioscorea tokoro* Makino.. J pharm Soc Japan.

[3] Correll DG, Schubert BG, Gentry HS, Hawley WO (1955). The search for plant precursors of cortisone.. Econ Bot.

[4] Applezweig N (1962). Steroid drugs.

[5] Miège J, Lyonga SN (1982). Yams-Ignames.

[6] Okonkwo SNC, Osuji GO (1985). The botany of the yam plant and its exploitation in enhanced productivity of the crop.. Advances in Yam Research.

[7] Lawton JRS, Lawton JR (1967). The morphology of the dormant embryo and young seedling of five species of *Dioscorea* in Nigeria.. Proc Linn Soc Lond.

[8] Degras L (1993). The Yam: A Tropical Root Crop.

[9] Smith BW (1937). Notes on the cytology and distribution of the Dioscoreaceae.. Bull Torr Bot Club.

[10] Ramachandran K (1968). Cytological studies in Dioscoreaceae.. Cytologia.

[11] Dahlgren G (1989). An undated angiosperm classification.. Botanical Journal of the Linnean Society.

[12] Zhang MZ, Wu ZJ, Qin HZ, Ding ZJ (1982). Comparative anatomy of Chinese *Dioscorea* and its meaning in sectional divisions.

[13] Su P (1987). Pollen morphology of *Dioscorea* in China.. Acta Phytotaxonomica Sinica.

[14] Qin HZ, Zhang MZ, Lin PP, Ding ZZ, Dou FP (1985). A Cytotaxonomic Study on Chinese *Dioscorea* L.—The chromosome numbers and their relation to the origin and evolution of the genus.. Acta Phytotaxonomica Sinica.

[15] Schols P, Furness CA, Wilkin P, Smets E, Cielen V (2003). Pollen morphology of *Dioscorea* (Dioscoreaceae) and its relation to systematics.. Botanical Journal of the Linnean Society.

[16] Schols P, Wilkin P, Furness CA, Huysmans S, Smets E (2005). Pollen evolution in Yams (*Dioscorea*: Dioscoreaceae).. Systematic botany.

[17] Kawabe A, Miyashita NT, Terauchi R (1997). Phylogenetic relationship among the section Stenophora in the genus *Dioscorea* based on the analysis of nucleotide sequence variation in the phosphoglucose isomerase (Pgi) locus.. Genes and Genetic Systems.

[18] Wilkin P, Schols P, Chase MW, Chayamarit K, Furness CA (2005). A plastid gene phylogeny of the yam genus, *Dioscorea*: roots, fruits and Madagascar.. Systematic Botany.

[19] Gao X, Zhu YP, Wu BC, Zhao YM, Chen JQ (2008). Phylogeny of *Dioscorea* sect. *Stenophora* based on chloroplast *mat*K, *rbc*L and *trn*L-F sequences.. Journal of Systematics and Evolution.

[20] Schindel DE, Miller SE (2005). DNA barcoding a useful tool for taxonomists.. Nature.

[21] Savolainen V, Cowan RS, Vogler AP, Roderick GK, Lane R (2005). Towards writing the encyclopedia of life: an introduction to DNA barcoding.. Philos Trans R Soc Lond B: Biol Sci.

[22] Marshall E (2005). Taxonomy. Will DNA bar codes breathe life into classification?. Science.

[23] Lahaye R, van der Bank M, Bogarin D, Warner J, Pupulin F (2008). DNA barcoding the floras of biodiversity hotspots.. Proc Natl Acad Sci U S A.

[24] Hebert PDN, Stoeckle MY, Zemlak TS, Francis CM (2004). Identification of birds through DNA barcodes.. PLoS Biol.

[25] Yoo HS, Eah JY, Kim JS, Kim YJ, Min MS (2006). DNA barcoding Korean birds.. Molecules and Cells.

[26] Ward RD, Zemlak TS, Innes BH, Last PR, Hebert PDN (2005). DNA barcoding Australia's fish species.. Philos Trans R Soc Lond B: Biol Sci.

[27] Yancy HF, Zemlak TS, Mason JA, Washington JD, Tenge BJ (2008). Potential use of DNA barcodes in regulatory science: Applications of the regulatory fish encyclopedia.. Journal of Food Protection.

[28] Hajibabaei M, Janzen DH, Burns JM, Hallwachs W, Hebert PDN (2006). DNA barcodes distinguish species of tropical *Lepidoptera*.. Proc Natl Acad Sci U S A.

[29] Hajibabaei M, Singer GAC, Clare EL, Hebert PDN (2007). Design and applicability of DNA arrays and DNA barcodes in biodiversity monitoring.. BMC Biol.

[30] Kress WJ, Wurdack KJ, Zimmer EA, Weigt LA, Janzen DH (2005). Use of DNA barcodes to identify flowering plants.. Proc Natl Acad Sci U S A.

[31] Kress WJ, Erickson DL (2007). A two-locus global DNA barcode for land plants: the coding rbcL gene complements the non-coding *trn*H-*psb*A spacer region.. PloS One.

[32] Kress WJ, Erickson DL (2008). DNA barcodes: Genes, genomics, and bioinformatics.. Proc Natl Acad Sci U S A.

[33] Thomas C (2009). Plant barcode soon to become reality.. Science.

[34] Burkill IH, Steenis CGGJvan (1951). Dioscoreaceae.. Flora Malesiana, Series I, Volume 4.

[35] Wan JR, Ding ZZ, Qin HZ (1994). A phytogeographical study on the family Dioscoreaceae.. Acta Botanica Boreale-Occidentalia Sinica.

[36] Meyer CP, Paulay G (2005). DNA barcoding: Error rates based on comprehensive sampling.. PLoS Biol.

[37] CBOL Plant Working Group (2009). A DNA barcode for land plants.. Proc Natl Acad Sci U S A.

[38] Sass C, Little DP, Stevenson DW, Specht CD (2007). DNA barcoding in the cycadales: testing the potential of proposed barcoding markers for species identification of cycads.. PloS One.

[39] Fazekas AJ, Burgess KS, Kesanakurti PR, Graham SW, Newmaster SG (2008). Multiple multilocus DNA barcodes from the plastid genome discriminate plant species equally well.. PloS One.

[40] Lahaye R, Savolainen V, Duthoit S, Maurin O, Bank Mvd (2008). A test of *psb*K-*psb*I and *atp*F-*atp*H as potential plant DNA barcodes using the flora of the Kruger National Park as a model system (South Africa).. http://hdl.handle.net/10101/npre.2008.1896.1.

[41] Newmaster SG, Fazekas AJ, Steeves RAD, Janovec J (2008). Testing candidate plant barcode regions in the Myristicaceae.. Molecular Ecology Resources.

[42] Newmaster SG, Fazekas AJ, Ragupathy S (2006). DNA barcoding in the land plants: evaluation of *rbc*L in a multigene tiered approach.. Canadian Journal of Botany.

[43] Whipple IG, Barkworth ME, Bushman BS (2007). Molecular insights into the taxonomy of *Glyceria* (Poaceae: Meliceae) in North America.. American Journal of Botany.

[44] Logacheva MD, Valiejo-Roman CM, Pimenov MG (2008). ITS phylogeny of West Asian *Heracleum* species and related taxa of Umbelliferae-Tordylieae W.D.J. Koch, with notes on evolution of their *psb*A-*trn*H sequences.. Plant Systematics and Evolution.

[45] Aldrich JBW, Cherney E, Merlin LC (1988). The role of insertions/deletions in the evolution of the intergenic region between *psb*A and *trn*H in the chloroplast genome.. Current Genetics.

[46] Mast AR, Givnish TJ (2002). Historical biogeography and the origin of stomatal distributions in *Banksia* and *Dryandra* (Proteaceae) based on their cpDNA phylogeny.. American Journal of Botany.

[47] Miller JT, Grimes JW, Murphy DJ, Bayer RJ, Ladiges PY (2003). A phylogenetic analysis of the Acacieae and Ingeae (Mimosoideae: Fabaceae) based on *trn*K, *mat*K, *psb*A-*trn*H, and *trn*L/*trn*F sequence data.. Systematic Botany.

[48] Kyndt T, Droogenbroeck BV, Romeijn-Peeters E, Romero-Motochi JP, Scheldeman X (2005). Molecular phylogeny and evolution of Caricaceae based on rDNA internal transcribed spacers and chloroplast sequence data.. Molecular Phylogenetics and Evolution.

[49] Shaw J, Lickey EB, Beck JT, Farmer SB, Liu W (2005). The tortoise and the hare II: relative utility of 21 noncoding chloroplast DNA sequences for phylogenetic analysis.. American Journal of Botany.

[50] Winkworth RC, Donoghue MJ (2005). Viburnum phylogeny based on combined molecular data: implications for taxonomy and biogeography.. American Journal of Botany.

[51] Huang CH, Hang YY, Zhou YF, Shi DR, Guo KY (2003). Population genetic structure of *Dioscorea zingiberensis* in China.. Acta Botanica Yunnanica.

[52] Chase MW, Cowan RS, Hollingsworth PM, van den Berg C, Madrinan S (2007). A proposal for a standardized protocol to barcode all land plants.. Taxon.

[53] Pennisi E (2007). Taxonomy: Wanted a barcode for plants.. Science.

[54] Hollingsworth PM (2008). DNA barcoding plants in biodiversity hot spots: Progress and outstanding questions.. Heredity.

[55] Chen SL, Yao H, Han JP, Liu C, Song JY (2010). Validation of the ITS2 region as a novel DNA barcode for identifying medicinal plant species.. PLoS ONE.

[56] Li DZ, Gao LM, Li HT, Wang H, China Plant BOL Group (2011). Comparative analysis of a large dataset indicates that internal transcribed spacer (ITS) should be incorporated into the core barcode for seed plants.. Proc Natl Acad Sci U S A.

[57] Paterson AH, Brubaber CL, Wendel JF (1993). A rapid method for extraction of cotton (Gossypium spp) genomic DNA suitable for RFLP of PCR analysis.. Plant Mol Bio Rep.

[58] Fay MF, Swensen SM, Chase MW (1997). Taxonomic affinities of *Medusagyne oppositifolia* (Medusagynaceae).. Kew Bulletin.

[59] Tate JA, Simpson BB (2003). Paraphyly of *Tarasa* (Malvaceae) and diverse origins of the polyploid species.. Systematic Botany.

[60] Meier R, Zhang GY, Ali F (2008). The use of mean instead of smallest interspecific distances exaggerates the size of the “Barcoding Gap” and leads to misidentification.. Syst Biol.

[61] Ross HA, Murugan S, Li WL (2008). Testing the reliability of genetic methods of species identification via simulation.. Syst Biol.

[62] Chase MW, Salamin N, Wilkinson M, Dunwell JM, Kesanakurthi RP (2005). Land plants and DNA barcodes: short-term and longterm goals.. Phil Trans R Soc B.

