# Supplementary material for: DNA Barcoding the Dioscorea in China, a Vital Group in the Evolution of Monocotyledon: Use of matK Gene for Species Discrimination
Source: PLoS One. 2012 Feb 20;7(2):e32057. doi: 10.1371/journal.pone.0032057 (PMC3282793; doi:10.1371/journal.pone.0032057)
Supplement: Table S1 — Wilcoxon two-sample tests for distribution of intra- vs. inter-specific divergences. (DOC) [file pone.0032057.s001.doc]

**Table S1. Wilcoxon two-sample tests for distribution of intra- vs. inter-specific divergences.**

| Region | Inter (N) | Intra (N) | Wilcoxon W | p-value |
| --- | --- | --- | --- | --- |
| *mat*K | 10501 | 377 | 503961.0 | 2.7879 × 10^-134^ |
| *rbc*L | 10501 | 377 | 803935.0 | 9.0795 × 10^-99^ |
| *psb*A*-trn*H | 10501 | 377 | 893513.5 | 2.5324 × 10^-83^ |
| *mat*K*+rbc*L | 10501 | 377 | 700540.5 | 1.7627 × 10^-112^ |
| *mat*K*+psb*A*-trn*H | 10501 | 377 | 820612.5 | 1.0885 × 10^-93^ |
| *rbc*L*+psb*A*-trn*H | 10501 | 377 | 735412.1 | 6.9201× 10^-114^ |
| *mat*K*+rbc*L*+psb*A*-trn*H | 10501 | 377 | 634703.0 | 2.6237 × 10^-124^ |
