# Supplementary material for: DNA Barcoding the Dioscorea in China, a Vital Group in the Evolution of Monocotyledon: Use of matK Gene for Species Discrimination
Source: PLoS One. 2012 Feb 20;7(2):e32057. doi: 10.1371/journal.pone.0032057 (PMC3282793; doi:10.1371/journal.pone.0032057)
Supplement: Table S2 — Samples for testing potential barcodes and accession numbers in GenBank. (DOC) [file pone.0032057.s002.doc]

**Table S2**. Samples for testing potential barcodes and accession numbers in GenBank.

| **Species** | **Section** | **Locality** | **Collection number** | **GenBank accession number** | | |
| --- | --- | --- | --- | --- | --- | --- |
|  |  |  |  | *mat*K | *rbc*L | *psb*A-*trn*H |
| *Dioscorea nipponica* Makino | Sect. Stenophora Uline | Lin’ an, Zhejiang, China | 200110112 | JQ259957 | JQ260105 | JQ260253 |
| *D. nipponica* Makino | Sect. Stenophora Uline | Wenchuan, Sichuan, China | 200708005 | JQ259958 | JQ260106 | JQ260254 |
| *D. nipponica* Makino | Sect. Stenophora Uline | Tianshui, Gansu, China | 200909005 | JQ259959 | JQ260107 | JQ260255 |
| *D. nipponica* Makino | Sect. Stenophora Uline | Ankang, Shanxi, China | 200909011 | JQ259960 | JQ260108 | JQ260256 |
| *D. nipponica* subsp. *rosthornii* Prain et Burkill | Sect. Stenophora Uline | Longnan, Gansu, China | 200909003 | JQ259961 | JQ260109 | JQ260257 |
| *D. nipponica* subsp. *rosthornii* Prain et Burkill | Sect. Stenophora Uline | Tianshui, Gansu, China | 200909004 | JQ259962 | JQ260110 | JQ260258 |
| *D. nipponica* subsp. *rosthornii* Prain et Burkill | Sect. Stenophora Uline | Enshi, Hubei, China | 200909019 | JQ259963 | JQ260111 | JQ260259 |
| *D. nipponica* subsp. *rosthornii* Prain et Burkill | Sect. Stenophora Uline | Hanzhong, Shanxi, China | 200909007 | JQ259964 | JQ260112 | JQ260260 |
| *D. nipponica* subsp. *rosthornii* Prain et Burkill | Sect. Stenophora Uline | Ankang, Shanxi, China | 200909012 | JQ259965 | JQ260113 | JQ260261 |
| *D. tokoro* Makino | Sect. Stenophora Uline | Anhua, Hunan, China | 200110122 | JQ259966 | JQ260114 | JQ260262 |
| *D. tokoro* Makino | Sect. Stenophora Uline | Enshi, Hubei, China | 200909021 | JQ259967 | JQ260115 | JQ260263 |
| *D. tokoro* Makino | Sect. Stenophora Uline | Wenzhou, Zhejiang, China | 200909055 | JQ259968 | JQ260116 | JQ260264 |
| *D. tokoro* Makino | Sect. Stenophora Uline | Nanping, Fujian, China | 200910003 | JQ259969 | JQ260117 | JQ260265 |
| *D. zingiberensis* C.H.Wight | Sect. Stenophora Uline | Hengshan, Hunan, China | 200211043 | JQ259970 | JQ260118 | JQ260266 |
| *D. zingiberensis* C.H.Wight | Sect. Stenophora Uline | Shiyan, Hubei, China | 200607002 | JQ259971 | JQ260119 | JQ260267 |
| *D. zingiberensis* C.H.Wight | Sect. Stenophora Uline | Ankang, Shanxi, China | NASHYY016 | JQ259972 | JQ260120 | JQ260268 |
| *D. zingiberensis* C.H.Wight | Sect. Stenophora Uline | Yichang, Sichuan, China | NASHYY017 | JQ259973 | JQ260121 | JQ260269 |
| *D. zingiberensis* C.H.Wight | Sect. Stenophora Uline | Liuba, Shanxi, China | NASHYY018 | JQ259974 | JQ260122 | JQ260270 |
| *D. zingiberensis* C.H.Wight | Sect. Stenophora Uline | Yingshan, Sichuan, China | NASHYY019 | JQ259975 | JQ260123 | JQ260271 |
| *D. zingiberensis* C.H.Wight | Sect. Stenophora Uline | Yunyang, Chongqing, China | NASHYY020 | JQ259976 | JQ260124 | JQ260272 |
| *D. zingiberensis* C.H.Wight | Sect. Stenophora Uline | Anhua, Hunan, China | NASHYY021 | JQ259977 | JQ260125 | JQ260273 |
| *D. zingiberensis* C.H.Wight | Sect. Stenophora Uline | Longnan, Gansu, China | 200909001 | JQ259978 | JQ260126 | JQ260274 |
| *D. zingiberensis* C.H.Wight | Sect. Stenophora Uline | Enshi, Hubei, China | 200909016 | JQ259979 | JQ260127 | JQ260275 |
| *D. parviflora* C.T.Ting | Sect. Stenophora Uline | Lijiang, Yunnan, China | 200211073 | JQ259980 | JQ260128 | JQ260276 |
| *D.* deltoidea Wallich ex Grisebach | Sect. Stenophora Uline | Diqing, Yunnan, China | 200709049 | JQ259981 | JQ260129 | JQ260277 |
| *D. panthaica* Prain & Burkill | Sect. Stenophora Uline | Lijiang, Yunnan, China | 200709257 | JQ259982 | JQ260130 | JQ260278 |
| *D. panthaica* Prain & Burkill | Sect. Stenophora Uline | Wenshan, Yunnan, China | GBOWS438 | JQ259983 | JQ260131 | JQ260279 |
| *D. biformifolia* C. Pei & C.T.Ting | Sect. Stenophora Uline | Yuxi, Yunnan, China | 200709174 | JQ259984 | JQ260132 | JQ260280 |
| *D. gracillima* Miquel | Sect. Stenophora Uline | Huangshan, Anhui, China | 200910001 | JQ259985 | JQ260133 | JQ260281 |
| *D. gracillima* Miquel | Sect. Stenophora Uline | Shangrao, Jiangxi, China | 200910002 | JQ259986 | JQ260134 | JQ260282 |
| *D. gracillima* Miquel | Sect. Stenophora Uline | Fuzhou, Jiangxi, China | 200910017 | JQ259987 | JQ260135 | JQ260283 |
| *D. gracillima* Miquel | Sect. Stenophora Uline | Jishou, Hunan, China | 200910028 | JQ259988 | JQ260136 | JQ260284 |
| *D. gracillima* Miquel | Sect. Stenophora Uline | Shaoyang, Hunan, China | 200910029 | JQ259989 | JQ260137 | JQ260285 |
| *D. gracillima* Miquel | Sect. Stenophora Uline | Lishui, Zhejiang, China | 200909050 | JQ259990 | JQ260138 | JQ260286 |
| *D. gracillima* Miquel | Sect. Stenophora Uline | Wenzhou, Zhejiang, China | 200909063 | JQ259991 | JQ260139 | JQ260287 |
| *D. gracillima* Miquel | Sect. Stenophora Uline | Nanping, Fujian, China | 200910009 | JQ259992 | JQ260140 | JQ260288 |
| *D. gracillima* Miquel | Sect. Stenophora Uline | Nanping, Fujian, China | 200910028 | JQ259993 | JQ260141 | JQ260289 |
| *D. gracillima* Miquel | Sect. Stenophora Uline | Huangshan, Anhui, China | 200910011 | JQ259994 | JQ260142 | JQ260290 |
| *D. collettii* J. D. Hooker | Sect. Stenophora Uline | Emeishan, Sichuan, China | 200308004 | JQ259995 | JQ260143 | JQ260291 |
| *D. collettii* J. D. Hooker | Sect. Stenophora Uline | Enshi, Hubei, China | 200909020 | JQ259996 | JQ260144 | JQ260292 |
| *D. collettii* J. D. Hooker | Sect. Stenophora Uline | Lishui, Zhejiang, China | 200909013 | JQ259997 | JQ260145 | JQ260293 |
| *D. collettii* J. D. Hooker | Sect. Stenophora Uline | Wenshan, Yunnan, China | GBOWS620 | JQ259998 | JQ260146 | JQ260294 |
| *D. collettii* var. *hypoglauca* (Palibin) C. T. Ting et al. | Sect. Stenophora Uline | Lishui, Zhejiang, China | 200909051 | JQ259999 | JQ260147 | JQ260295 |
| *D. collettii* var. *hypoglauca* (Palibin) C. T. Ting et al. | Sect. Stenophora Uline | Wenzhou, Zhejiang, China | 200909060 | JQ260000 | JQ260148 | JQ260296 |
| *D. collettii* var. *hypoglauca* (Palibin) C. T. Ting et al. | Sect. Stenophora Uline | Fuzhou, Jiangxi, China | 200910011 | JQ260001 | JQ260149 | JQ260297 |
| *D. collettii* var. *hypoglauca* (Palibin) C. T. Ting et al. | Sect. Stenophora Uline | Huangshan, Anhui, China | 200910009 | JQ260002 | JQ260150 | JQ260298 |
| *D. futschauensis* Uline ex R. Knuth | Sect. Stenophora Uline | Fuzhou, Fujian, China | 200211015 | JQ260003 | JQ260151 | JQ260299 |
| *D. futschauensis* Uline ex R. Knuth | Sect. Stenophora Uline | Fuzhou, Fujian, China | 200211020 | JQ260004 | JQ260152 | JQ260300 |
| *D. septemloba* Thunb. | Sect. Stenophora Uline | Hengyang, Hunan, China | 200211042 | JQ260005 | JQ260153 | JQ260301 |
| *D. septemloba* Thunb. | Sect. Stenophora Uline | Wenzhou, Zhejiang, China | 200909057 | JQ260006 | JQ260154 | JQ260302 |
| *D. tenuipes* Franchet & Savatier | Sect. Stenophora Uline | Lishui, Zhejiang, China | 200909002 | JQ260007 | JQ260155 | JQ260303 |
| *D. banzhuana* C. P’ei & C. T. Ting | Sect. Stenophora Uline | Honghe, Yunnan, China | 200212106 | JQ260008 | JQ260156 | JQ260304 |
| *D. simulans* Prain & Burkill | Sect. Stenophora Uline | Guilin, Guangxi, China | 200211047 | JQ260009 | JQ260157 | JQ260305 |
| *D.* esculenta (Loureiro) Burkill | Sect. Combilium Prain et Burkill | Haikou, Hainan, China | 200804021 | JQ260010 | JQ260158 | JQ260306 |
| *D. esculenta* var. *spinosa* (Roxburgh ex Prain & Burkill) R. Knuth | Sect. Combilium Prain et Burkill | Haikou, Hainan, China | 200804023 | JQ260011 | JQ260159 | JQ260307 |
| *D. tentaculigera* Prain & Burkill | Sect. Shannieorea Prain et Burkill | Lincang, Yunnan, China | 200709074 | JQ260012 | JQ260160 | JQ260308 |
| *D. subcalva* Prain & Burkill | Sect. Shannieorea Prain et Burkill | Baise, Guangxi, China | 200211071 | JQ260013 | JQ260161 | JQ260309 |
| *D. subcalva* Prain & Burkill | Sect. Shannieorea Prain et Burkill | Gejiu, Yunnan, China | 200212103 | JQ260014 | JQ260162 | JQ260310 |
| *D. subcalva* Prain & Burkill | Sect. Shannieorea Prain et Burkill | Guiyang, Guizhou, China | 200709195 | JQ260015 | JQ260163 | JQ260311 |
| *D. subcalva* Prain & Burkill | Sect. Shannieorea Prain et Burkill | Lincang, Yunnan, China | 200808208 | JQ260016 | JQ260164 | JQ260312 |
| *D. subcalva* var. *submollis* (R. Knuth) C. T. Ting & P. P. Ling | Sect. Shannieorea Prain et Burkill | Nanchuan, Chongqing, China | 200308028 | JQ260017 | JQ260165 | JQ260313 |
| *D. nitens* Prain & Burkill | Sect. Shannieorea Prain et Burkill | Lijiang, Yunnan, China | 200211080 | JQ260018 | JQ260166 | JQ260314 |
| *D. nitens* Prain & Burkill | Sect. Shannieorea Prain et Burkill | Kunming, Yunnan, China | 200709006 | JQ260019 | JQ260167 | JQ260315 |
| *D. nitens* Prain & Burkill | Sect. Shannieorea Prain et Burkill | Yuxi, Yunnan, China | 200709171 | JQ260020 | JQ260168 | JQ260316 |
| *D. bulbifera* Linnaeus | Sect. Opsophyton Mine | Shaoguan, Guangdong, China | 200911007 | JQ260021 | JQ260169 | JQ260317 |
| *D. bulbifera* Linnaeus | Sect. Opsophyton Mine | Wuyishan, Fujian, China | 200910001 | JQ260022 | JQ260170 | JQ260318 |
| *D. bulbifera* Linnaeus | Sect. Opsophyton Mine | Liu’an, Anhui, China | 200910009 | JQ260023 | JQ260171 | JQ260319 |
| *D. bulbifera* Linnaeus | Sect. Opsophyton Mine | Emeishan, Sichuan, China | 200308001 | JQ260024 | JQ260172 | JQ260320 |
| *D. bulbifera* Linnaeus | Sect. Opsophyton Mine | Hengyang, Hunan, China | 200211038 | JQ260025 | JQ260173 | JQ260321 |
| *D. bulbifera* Linnaeus | Sect. Opsophyton Mine | Congzuo, Guangxi, China | 200211059 | JQ260026 | JQ260174 | JQ260322 |
| *D. bulbifera* Linnaeus | Sect. Opsophyton Mine | Wuhu, Anhui, China | 200510003 | JQ260027 | JQ260175 | JQ260323 |
| *D. bulbifera* Linnaeus | Sect. Opsophyton Mine | Lishui, Zhejiang, China | 200909023 | JQ260028 | JQ260176 | JQ260324 |
| *D. bulbifera* Linnaeus | Sect. Opsophyton Mine | Shangrao, Jiangxi, China | 200910003 | JQ260029 | JQ260177 | JQ260325 |
| *D. bulbifera* Linnaeus | Sect. Opsophyton Mine | Shaoguan, Guangdong, China | 200211037 | JQ260030 | JQ260178 | JQ260326 |
| *D. bulbifera* Linnaeus | Sect. Opsophyton Mine | Haikou, Hainan, China | 200309013 | JQ260031 | JQ260179 | JQ260327 |
| *D. bulbifera* Linnaeus | Sect. Opsophyton Mine | Jinghong, Yunnan, China | 200709142 | JQ260032 | JQ260180 | JQ260328 |
| *D. bulbifera* Linnaeus | Sect. Opsophyton Mine | Honghe, Yunnan, China | GBOWS788 | JQ260033 | JQ260181 | JQ260329 |
| *D. kamoonensis* Kunth | Sect. Lasiophyton Uline | Kunming, Yunnan, China | 2002121070 | JQ260034 | JQ260182 | JQ260330 |
| *D. kamoonensis* Kunth | Sect. Lasiophyton Uline | Huaxi, Guizhou, China | 200709256 | JQ260035 | JQ260183 | JQ260331 |
| *D. kamoonensis* Kunth | Sect. Lasiophyton Uline | Wenshan, Yunnan, China | GBOWS556 | JQ260036 | JQ260184 | JQ260332 |
| *D. delavayi* Franchet | Sect. Lasiophyton Uline | Kunming, Yunnan, China | 200709040 | JQ260037 | JQ260185 | JQ260333 |
| *D. menglaensis* H. Li | Sect. Lasiophyton Uline | Jinghong, Yunnan, China | 200709160 | JQ260038 | JQ260186 | JQ260334 |
| *D.menglaensis* H. Li | Sect. Lasiophyton Uline | Lincang, Yunnan, China | 200808205 | JQ260039 | JQ260187 | JQ260335 |
| *D. pentaphylla* Linnaeus | Sect. Lasiophyton Uline | Jinghong, Yunnan, China | 200212089 | JQ260040 | JQ260188 | JQ260336 |
| *D. pentaphylla* Linnaeus | Sect. Lasiophyton Uline | Lincang, Yunnan, China | 200709060 | JQ260041 | JQ260189 | JQ260337 |
| *D. pentaphylla* Linnaeus | Sect. Lasiophyton Uline | Congzuo, Guangxi, China | 200709218 | JQ260042 | JQ260190 | JQ260338 |
| *D. pentaphylla* Linnaeus | Sect. Lasiophyton Uline | Qingyuan, Guangdong, China | 200911017 | JQ260043 | JQ260191 | JQ260339 |
| *D. esquirolii* Prain & Burkill | Sect. Lasiophyton Uline | Honghe, Yunnan, China | GBOWS1205 | JQ260044 | JQ260192 | JQ260340 |
| *D. hispida* Dennstedt | Sect. Lasiophyton Uline | Congzuo, Guangxi, China | 200211061 | JQ260045 | JQ260193 | JQ260341 |
| *D. hispida* Dennstedt | Sect. Lasiophyton Uline | Haikou, Hainan, China | 200309018 | JQ260046 | JQ260194 | JQ260342 |
| *D. aspersa* Prain & Burkill | Sect. Enantiophyllum Uline | Gejiu, Yunnan, China | 200212109 | JQ260047 | JQ260195 | JQ260343 |
| *D. polystachya* Turczaninow | Sect. Enantiophyllum Uline | Zhenjiang, Jiangsu, China | 200110125 | JQ260048 | JQ260196 | JQ260344 |
| *D. polystachya* Turczaninow | Sect. Enantiophyllum Uline | Wenchuan, Sichuan, China | 200308017 | JQ260049 | JQ260197 | JQ260345 |
| *D. polystachya* Turczaninow | Sect. Enantiophyllum Uline | Nanchuan, Chongqing, China | 200308029 | JQ260050 | JQ260198 | JQ260346 |
| *D. polystachya* Turczaninow | Sect. Enantiophyllum Uline | Lijiang, Yunnan, China | 200709045 | JQ260051 | JQ260199 | JQ260347 |
| *D. polystachya* Turczaninow | Sect. Enantiophyllum Uline | Yantai, Shandong, China | NASHYY096 | JQ260052 | JQ260200 | JQ260348 |
| *D. polystachya* Turczaninow | Sect. Enantiophyllum Uline | Jiaozuo, Henan, China | 200410101 | JQ260053 | JQ260201 | JQ260349 |
| *D. polystachya* Turczaninow | Sect. Enantiophyllum Uline | Liu’an, Anhui, China | 200910003 | JQ260054 | JQ260202 | JQ260350 |
| *D. polystachya* Turczaninow | Sect. Enantiophyllum Uline | Shanming, Fujian, China | 200910023 | JQ260055 | JQ260203 | JQ260351 |
| *D. polystachya* Turczaninow | Sect. Enantiophyllum Uline | Longnan, Gansu, China | 200909002 | JQ260056 | JQ260204 | JQ260352 |
| *D. polystachya* Turczaninow | Sect. Enantiophyllum Uline | Hanzhong, Shanxi, China | 200909006 | JQ260057 | JQ260205 | JQ260353 |
| *D. polystachya* Turczaninow | Sect. Enantiophyllum Uline | Lishui, Zhejiang, China | 200909026 | JQ260058 | JQ260206 | JQ260354 |
| *D. japonica* Thunberg | Sect. Enantiophyllum Uline | Shangrao, Jiangxi, China | 200910001 | JQ260059 | JQ260207 | JQ260355 |
| *D. japonica* Thunberg | Sect. Enantiophyllum Uline | Jishou, Hunan, China | 200910021 | JQ260060 | JQ260208 | JQ260356 |
| *D. japonica* Thunberg | Sect. Enantiophyllum Uline | Qingyuan, Guangdong, China | 200911020 | JQ260061 | JQ260209 | JQ260357 |
| *D. japonica* Thunberg | Sect. Enantiophyllum Uline | Wuyishan, Fujian, China | 200910002 | JQ260062 | JQ260210 | JQ260358 |
| *D. japonica* Thunberg | Sect. Enantiophyllum Uline | Liu’an, Anhui, China | 200910001 | JQ260063 | JQ260211 | JQ260359 |
| *D. japonica* var. *pilifera* C. T. Ting & M. C. Chang | Sect. Enantiophyllum Uline | Fuzhou, Jiangxi, China | 200910008 | JQ260064 | JQ260212 | JQ260360 |
| *D. japonica* var. *pilifera* C. T. Ting & M. C. Chang | Sect. Enantiophyllum Uline | Shaoyang, Hunan, China | 200910032 | JQ260065 | JQ260213 | JQ260361 |
| *D. cirrhosa* Loureiro | Sect. Enantiophyllum Uline | Lincang, Yunnan, China | 200808203 | JQ260066 | JQ260214 | JQ260362 |
| *D. cirrhosa* Loureiro | Sect. Enantiophyllum Uline | Jinghong, Yunnan, China | 200709154 | JQ260067 | JQ260215 | JQ260363 |
| *D. cirrhosa* Loureiro | Sect. Enantiophyllum Uline | Meizhou, Guangdong, China | 200911005 | JQ260068 | JQ260216 | JQ260364 |
| *D. cirrhosa* Loureiro | Sect. Enantiophyllum Uline | Binzhou, Hunan, China | 200910039 | JQ260069 | JQ260217 | JQ260365 |
| *D. cirrhosa* Loureiro | Sect. Enantiophyllum Uline | Fuzhou, Jiangxi, China | 200910019 | JQ260070 | JQ260218 | JQ260366 |
| *D. cirrhosa* Loureiro | Sect. Enantiophyllum Uline | Lishui, Zhejiang, China | 200909048 | JQ260071 | JQ260219 | JQ260367 |
| *D. cirrhosa* Loureiro | Sect. Enantiophyllum Uline | Wenshan, Yunnan, China | GBOWS465 | JQ260072 | JQ260220 | JQ260368 |
| *D. cirrhosa* Loureiro | Sect. Enantiophyllum Uline | Wenshan, Yunnan, China | GBOWS522 | JQ260073 | JQ260221 | JQ260369 |
| *D. cirrhosa* Loureiro | Sect. Enantiophyllum Uline | Wenshan, Yunnan, China | GBOWS557 | JQ260074 | JQ260222 | JQ260370 |
| *D. cirrhosa* var. *cylindrica* C. T. Ting & M. C. Chang | Sect. Enantiophyllum Uline | Meishou, Guangdong, China | 200911006 | JQ260075 | JQ260223 | JQ260371 |
| *D. glabra* Roxburgh | Sect. Enantiophyllum Uline | Xishuangbanna, Yunnan, China | 200709102 | JQ260076 | JQ260224 | JQ260372 |
| *D. glabra* Roxburgh | Sect. Enantiophyllum Uline | Jinghong, Yunnan, China | 200709156 | JQ260077 | JQ260225 | JQ260373 |
| *D. glabra* Roxburgh | Sect. Enantiophyllum Uline | Enshi, Hubei, China | 200909015 | JQ260078 | JQ260226 | JQ260374 |
| *D. fordii* Prain & Burkill | Sect. Enantiophyllum Uline | Haikou, Hainan, China | 200309012 | JQ260079 | JQ260227 | JQ260375 |
| *D. fordii* Prain & Burkill | Sect. Enantiophyllum Uline | Haikou, Hainan, China | 200309010 | JQ260080 | JQ260228 | JQ260376 |
| *D. fordii* Prain & Burkill | Sect. Enantiophyllum Uline | Lishui, Zhejiang, China | 200909053 | JQ260081 | JQ260229 | JQ260377 |
| *D. fordii* Prain & Burkill | Sect. Enantiophyllum Uline | Qingyuan, Guangdong, China | 200911018 | JQ260082 | JQ260230 | JQ260378 |
| *D. persimilis* Prain & Burkill | Sect. Enantiophyllum Uline | Shanming, Fujian, China | 200410201 | JQ260083 | JQ260231 | JQ260379 |
| *D. persimilis* Prain & Burkill | Sect. Enantiophyllum Uline | Jishou, Hunan, China | 200910025 | JQ260084 | JQ260232 | JQ260380 |
| *D. persimilis* Prain & Burkill | Sect. Enantiophyllum Uline | Meizhou, Guangdong, China | 200911004 | JQ260085 | JQ260233 | JQ260381 |
| *D. persimilis* Prain & Burkill | Sect. Enantiophyllum Uline | Qingyuan, Guangdong, China | 200911016 | JQ260086 | JQ260234 | JQ260382 |
| *D. persimilis* Prain & Burkill | Sect. Enantiophyllum Uline | Nanping, Fujian, China | 200910034 | JQ260087 | JQ260235 | JQ260383 |
| *D. persimilis* var. *pubescens* C. T. Ting & M. C. Chang | Sect. Enantiophyllum Uline | Jishou, Hunan, China | 200910024 | JQ260088 | JQ260236 | JQ260384 |
| *D. exalata* C. T. Ting & M. C. Chang | Sect. Enantiophyllum Uline | Baise, Guangxi, China | 200211068 | JQ260089 | JQ260237 | JQ260385 |
| *D. exalata* C. T. Ting & M. C. Chang | Sect. Enantiophyllum Uline | Yuxi, Yunnan, China | Y172 | JQ260090 | JQ260238 | JQ260386 |
| *D. exalata* C. T. Ting & M. C. Chang | Sect. Enantiophyllum Uline | Guilin, Guangxi, China | 200211047 | JQ260091 | JQ260239 | JQ260387 |
| *D. exalata* C. T. Ting & M. C. Chang | Sect. Enantiophyllum Uline | Kunming, Yunnan, China | 200511003 | JQ260092 | JQ260240 | JQ260388 |
| *D. exalata* C. T. Ting & M. C. Chang | Sect. Enantiophyllum Uline | Haikou, Hainan, China | 200804027 | JQ260093 | JQ260241 | JQ260389 |
| *D. exalata* C. T. Ting & M. C. Chang | Sect. Enantiophyllum Uline | Qingyuan, Guangdong, China | 200911019 | JQ260094 | JQ260242 | JQ260390 |
| *D. alata* Linnaeus | Sect. Enantiophyllum Uline | Jinghong, Yunnan, China | 200212093 | JQ260095 | JQ260243 | JQ260391 |
| *D. alata* Linnaeus | Sect. Enantiophyllum Uline | Haikou, Hainan, China | 200309002 | JQ260096 | JQ260244 | JQ260392 |
| *D. alata* Linnaeus | Sect. Enantiophyllum Uline | Zhejiang, China | 200410202 | JQ260097 | JQ260245 | JQ260393 |
| *D. alata* Linnaeus | Sect. Enantiophyllum Uline | Taiwan, China | 200511018 | JQ260098 | JQ260246 | JQ260394 |
| *D. alata* Linnaeus | Sect. Enantiophyllum Uline | Meizhou, Guangdong , China | 200911001 | JQ260099 | JQ260247 | JQ260395 |
| *D. decipiens* J. D. Hooker | Sect. Enantiophyllum Uline | Jinghong, Yunnan, China | 200212095 | JQ260100 | JQ260248 | JQ260396 |
| *D. decipiens* J. D. Hooker | Sect. Enantiophyllum Uline | Lincang, Yunnan, China | 200709051 | JQ260101 | JQ260249 | JQ260397 |
| *D. decipiens* J. D. Hooker | Sect. Enantiophyllum Uline | Lincang, Yunnan, China | 200709051 | JQ260102 | JQ260250 | JQ260398 |
| *D. decipiens* J. D. Hooker | Sect. Enantiophyllum Uline | Lincang, Yunnan, China | 200709051 | JQ260103 | JQ260251 | JQ260399 |
| *D. decipiens* var. *glabrescens* C. T. Ting & M. C. Chang | Sect. Enantiophyllum Uline | Lincang, Yunnan, China | 200709098 | JQ260104 | JQ260252 | JQ260400 |
