# Supplementary material for: DNA Barcoding the Dioscorea in China, a Vital Group in the Evolution of Monocotyledon: Use of matK Gene for Species Discrimination
Source: PLoS One. 2012 Feb 20;7(2):e32057. doi: 10.1371/journal.pone.0032057 (PMC3282793; doi:10.1371/journal.pone.0032057)
Supplement: Table S3 — Primers and reaction conditions used in the study. (DOC) [file pone.0032057.s003.doc]

**Table S3. Primers and reaction conditions used in the study.**

| Locus | Name of primer | Primer sequence 5’-3’ | Annealing temperature in PCR^1^ | Resource |
| --- | --- | --- | --- | --- |
| *mat*K | int F | GGA TTT TCC GTC CAC CCT AT | 55℃ | RBG Edinbergh recommened |
|  | int R | TTA GCC GCA CAT TTG AAA AA |  |  |
|  | 3F_Kim | CGT ACA GTA CTT TTG TGT TTA CGA G | 55℃ | CBOL (http://barcoding.si.edu) |
|  | 1R_KIM | ACC CAG TCC ATC TGG AAA TCT TGG TTC |  |  |
|  | MF | ATT TGC GAT CTA TTC ATT CAA T | 58℃ | designed by ourselves |
|  | MR | TGA GAT TCC GCA GGT CAT T |  |  |
| *rbc*L | 1F | ATG TCA CCA CAA ACA GAA AC | 53℃ | Fay et al. 1997 |
|  | 724R | TCG CAT GTA CCT GCA GTA GC |  |  |
|  | 1Fm | ATG TCA CCA CAA ACA GAG AC | 55℃ | designed by ourselves |
|  | 724Rm  1163Rm | TCA CAT GTA CCC GCA GTA GC  AAA GAT TTC GGT CAG AGC AG |  |  |
| *psb*A-*trn*H | psbAF | GTT ATG CAT GAA CGT AAT GCT C | 55℃ | Tate & Simpson 2003 |
|  | trnH2 | CGC GCA TGG TGG ATT CAC AAT CC |  |  |
|  | *psb*A F1 | AAT GCT CAC AAC TTC CCT CTA | 56℃ | designed by ourselves |
|  | *trn*H R1 | CCA CTG CCT TGA TCC ACT TG |  |  |

^1^Polymerase chain reaction (PCR) amplifications of the three candidate barcode were carried out with the following program: a premelt of 3 min at 94℃, followed by 35 cycles of 45 s denaturation at 94℃, 30 s annealing at 53-58℃ (varied by different barcodes), 1.5 min 30 s extension at 72℃.
